# The Terrible Imposture and Force of Words

**Published:** 1903-11-21

**Authors:** 


### The Hospital.

A Journal of

The Medical Sciences and Hospital Administration.

Vol. XXXV.—No. 895. NOVEMBER 21, 1903.

### "The Terrible Imposture and Force of Words."

Such was the title selected by Dr. South for one of the many sermons, compositions of well-nigh Shakespearian power and beauty, by which he almost succeeded in touching the cold hearts, and in awakening the callous consciences, of the courtiers of Charles the Second; and the lesson which he desired to teach is one that has afforded subject matter to many writers since his day. In the course of some almost touching efforts to joke with difficulty, in the presence of serious questions, a few prominent politicians have lately endeavoured, not always with the best success, to apply medical technicalities to matters of public discussion. Mr. Asquith, for example, has declared the nation to be suffering from "fiscalitis," and the *Daily News*, from which he might have expected better treatment, but which possibly was unable to see its way to the inflammation of an adjective, printed his poor little joke "fiscalities," and so caused it to perish miserably in the first moments of its existence. We have, notwithstanding, seen other abortive forms of the suffix in various political paragraphs, and their appearance suggests the question whether the profession might not, with no small advantage, abstain from the use of language which is often as badly understood by those who use it as by those to whom it is addressed, and which may not only serve to obscure the truth, but sometimes to create a totally erroneous impression of its nature. Oliver Twist was duly initiated into the mysteries of a vocabulary which aimed at concealing the dealings of the law with criminality; and it is equally possible to conceal, under an unintelligible nomenclature professedly derived from classic tongues, the actual ugliness of conditions brought about by vicious indulgence. It may be questioned whether a general abandonment of this practice, on the part of physicians and surgeons, would be conducive to the immediate popularity of the doctors concerned; but it would certainly be conducive to a more correct estimate of the character and tendencies of several not uncommon practices. The nature of the effects of excessive drinking seems to be half concealed under the euphemistic "alcoholism," and the still more degrading drunkenness produced by cocaine or morphia appears to be comparatively innocent when it is regarded as the consequence of a "habit." It is recorded of Quin, the actor, whose gormandising propensities were notorious, that a lady to whom he was introduced received him with the words, "I hear, Mr. Quin, that you are a great epicure." "No, madam, replied Quin, "I am only a great glutton." It is more than probable that the majority of ailments in both sexes, which polite physicians describe as "indigestion," and the superlatively polite as "dyspepsia," would be put far more nearly into their right places in the public estimation if Quin's simple phraseology were systematically applied to them. Experience seems to teach, moreover, that doctors given to plain speaking have frequently succeeded in winning and in holding a very large share of the confidence of the public; as, for example, in the well-known cases of Abernethy and of Jepson, but it may perhaps be assumed that for this purpose some special gifts of character or manner would be essential. It is at least certain that designed imitations of these distinguished men have not always been rewarded by the success which they may possibly have deserved.

On the correctness of the general principle, however, that an increased plainness of speech would be conducive to a better understanding of illness on the part of the public, and to an increased avoidance of its causes, there can, we think, be but small question. It would not be necessary to go as far as the author of "Erewhon," and to anticipate the arrival of a state of society in which what we now call illness would be punished as crime, and what we now call crime would be regarded as misfortune, the consequence of heredity or of circumstance; but it is none the less desirable that we should endeavour to strip off many veils of circumlocution under which the true character of a vast amount of illness is at least partially concealed, and should thus endeavour to put both the illness itself, and the particular form of folly or of self-indulgence which has produced it, in a light somewhat less misleading than that which is now commonly employed. For a vast amount of misplaced sentimentality concerning illness we are, of course, indebted to foolish writers of fiction; and it is very largely on account of the maladies described in novels, and described in such terms that it would be very difficult for a physician to speak at all confidently concerning their nature, that the general "nastiness" of being ill, to put the matter plainly, has become somewhat obscured by pictures of interesting invalids and of ministering angels. There are, of course, exceptional cases of noble self-sacrifice or of heroic endurance; but the average man or woman who is ill is simply a person who has done something which he or she should have had sufficient common sense to avoid, and who is paying the penalty which Nature always exacts from those who violate her ordinances. "Nature," the late Sir Andrew Clark was wont to say, "never forgets and seldom forgives," and before her tribunal, as before that of the law, the plea of ignorance is unavailing. It is our duty to know and to follow the lines of action along which, barring what may be called accidents, our health will be preserved, and to know and to avoid those along which it is in danger of being impaired. To the diffusion of such knowledge, plainness of speech would be eminently conducive, and not the least of its merits would be that it would have to be in the vernacular. Nothing would add so much to the influence of the medical profession as the cultivation by its members of the power to express themselves in English. Mr. Asquith may be taken to have proved that the various terminations in "*itis*," when used instead of their equivalents in the tongue of Shakespeare and of Milton, convey very little meaning to many of those who employ them. Our own objection to them is more deeply rooted, and depends upon the profound illiteracy by which they are generally characterised. A scholar who heard a doctor talk about "appendicitis" or "iridectomy" would probably be too polite openly to deride the speaker, but he would smile internally, much as the doctor himself would smile at the attempted fine language of a charwoman. And the estimate of a learned profession which is formed by scholars must in due time percolate to the less instructed members of the community.

